# *PPARG* Epigenetic Deregulation and Its Role in Colorectal Tumorigenesis

**DOI:** 10.1155/2012/687492

**Published:** 2012-07-16

**Authors:** Lina Sabatino, Alessandra Fucci, Massimo Pancione, Vittorio Colantuoni

**Affiliations:** Department of Biological, Geological and Environmental Sciences, University of Sannio, via Port'Arsa 11, 82100 Benevento, Italy

## Abstract

Peroxisome proliferator-activated receptor gamma (PPAR**γ**) plays critical roles in lipid storage, glucose metabolism, energy homeostasis, adipocyte differentiation, inflammation, and cancer. Its function in colon carcinogenesis has largely been debated; accumulating evidence, however, supports a role as tumor suppressor through modulation of crucial pathways in cell differentiation, apoptosis, and metastatic dissemination. Epigenetics adds a further layer of complexity to gene regulation in several biological processes. In cancer, the relationship with epigenetic modifications has provided important insights into the underlying molecular mechanisms. These studies have highlighted how epigenetic modifications influence *PPARG* gene expression in colorectal tumorigenesis. In this paper, we take a comprehensive look at the current understanding of the relationship between PPAR**γ** and cancer development. The role that epigenetic mechanisms play is also addressed disclosing novel crosstalks between *PPARG* signaling and the epigenetic machinery and suggesting how this dysregulation may contribute to colon cancer development.

## 1. Introduction

Peroxisome-proliferator activated receptors (PPARs) are ligand-dependent transcription factors belonging to the nuclear receptor superfamily. Three PPAR isotypes have been identified so far: PPAR*α*  (NR1C1), PPAR*β*/*δ* (NR1C2), and PPAR*γ* (NR1C3), each displaying a tissue specific expression pattern. PPAR*α* is predominantly expressed in liver, brown adipose tissue, skeletal muscle, endothelium, and heart; PPAR*β*/*δ* has a broader expression pattern; PPAR*γ* is expressed in adipose tissue, muscle, gastrointestinal tract, blood cells, macrophages, and liver [1–3]. PPARs form permissive heterodimers with the retinoid X receptors (RXR) and recognize specific sequence motifs, defined PPRE (Peroxisome proliferator response elements), in the regulatory regions of target genes [3–6]. In the absence of ligand, PPARs are complexed with corepressor proteins such as NCoR (nuclear receptor corepressor) or SMRT (silencing mediator of retinoid and thyroid receptors) and act as transcriptional repressors. Ligand binding induces conformational changes that allow displacement of the corepressor complexes and recruitment of transcriptional coactivators. These include members of the steroid receptor coactivator (SRC) family and histone acetyltransferases, such as p300/CBP, that modify the chromatin structure at PPRE-containing promoters, affecting gene transcription [3, 7]. A variety of endogenous and exogenous compounds, including lipophilic molecules such as polyunsaturated fatty acids, prostaglandines, leukotrienes, and hypolipidemic drugs, have been identified as PPAR ligands. The structural heterogeneity of these ligands seems to reflect the conformation of the ligand binding domain (LBD), which forms a large Y-shaped hydrophobic pocket with relatively low ligand specificity [8].

**Figure 1:**
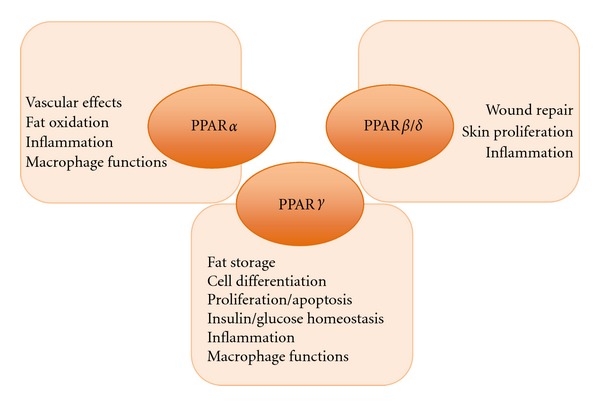
Overview of PPARs physiological roles.

PPARs modulate cellular and whole-body glucose and lipid homeostasis. Upon activation by the synthetic agonists fenofibrate and gemfibrozil, PPAR*α* stimulates hepatic lipid uptake and catabolism displaying antiatherosclerotic and hypolipidemic effects. PPAR*γ* is activated by the antidiabetic agents thiazolidinediones (TZDs) and increases insulin sensitivity in adipose and muscle tissues. Genetic and pharmacological studies have revealed important roles of PPAR*β*/*δ* in regulating lipid metabolism and energy homeostasis [8, 9]. In addition to their metabolic effects, PPARs have also been implicated in the modulation of immune and inflammatory processes, vascular homeostasis, tissue remodeling, cell differentiation, and proliferation both in normal and neoplastic tissues (Figure 1) [10–16]. In recent years, several studies have addressed the role of PPARs in cancer development. PPAR*α* has shown tumor-promoting effects in rodents inducing hepatocarcinoma formation. Its role in humans is less clear but its activation by exogenous agonists causes inhibition of tumor cell growth in cell lines derived from different tumors [16–20]. Conflicting data have suggested a role for PPAR*β*/*δ* either as a tumor suppressor or as a tumor promoter [21–24]. Finally, a large body of evidence supports PPAR*γ* involvement in tumor development. 

## 2. *PPARG *Structure and Function 

**Figure 2:**
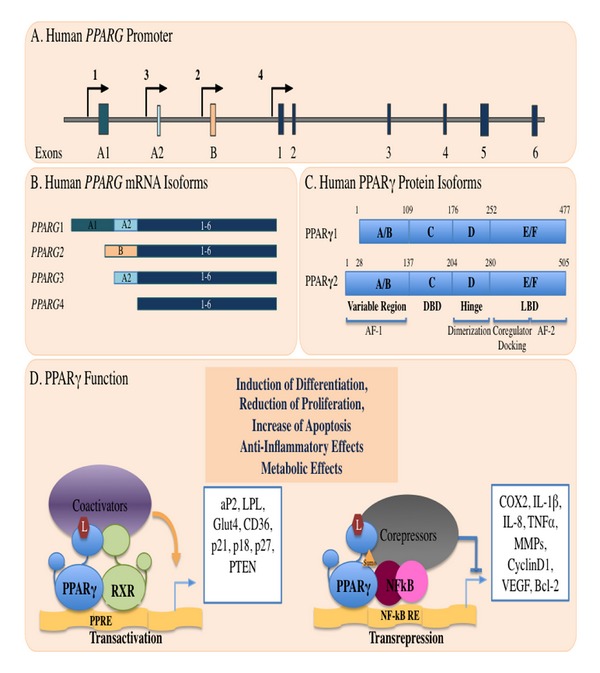
(a) *PPARG* schematic structure at chromosome 3p25. The arrows indicate the transcription start sites for each specific mRNA isoform; the boxes indicate the exons. (b) The four mature transcribed mRNAs are depicted. (c) The *PPARG*1, 3, and 4 mRNA isoforms are translated into the unique PPAR*γ*1 protein; the *PPARG*2 transcript is translated into PPAR*γ*2, containing 28 additional amino acids at its N-terminal region. The four functional domains of the mature protein are reported. (d) PPAR*γ* mechanisms of action. Transactivation: in the presence of ligands, PPAR*γ* binds the cognate PPRE as heterodimer with RXR and activates gene expression. Transrepression: in the presence of ligands, the SUMOylated form of the receptor interacts with others transcription factors, such as NF*κ*B, and represses their target genes transcription, adapted from reference [26].

*PPARG *is located on human chromosome 3p25.2, spans a region of 100 Kb in length, and is organized in nine exons. Four major transcriptional start sites have been identified and, by differential promoter usage and alternative splicing, four mature mRNAs are generated differing in their 5′ end. Three transcripts, *PPARG*1, 3, and 4, produce the identical protein PPAR*γ*1. *PPARG*2 transcript, in contrast, uses a different translational start codon and synthesizes PPAR*γ*2 with 28 additional amino acids at the N-terminus (Figure 2) [25–29]. The mature protein shares the same overall structure of all nuclear receptors. The A/B region at the N-terminus is the most variable in length and sequence and is the key determinant of isotype-selective gene expression and function [30]. It contains the ligand-independent transactivation domain AF1, (residues 1–71 of PPAR*γ*1) the lysine 79 and serine 84 residues, targets of SUMOylation, and phosphorylation events, respectively, that negatively regulate receptor activity [31]. The C region is the DNA binding domain, characterized by two C4 zinc-finger motifs, that interact with the major groove of the DNA. The D or hinge region allows receptor dimerization and DNA binding. The E/F region is the ligand binding domain (LBD) constituted by 12 *α* helices where the agonist accommodates. Ligand addition induces structural changes in the LBD that enable corepressors release and coactivators recruitment, mainly through the AF2 domain in helix 12, entitling ligand-dependent transactivation [32, 33]. PPAR*γ*1 and *γ*2 isoforms have a cell-specific expression pattern, although the functional differences have not been completely elucidated [34]. PPAR*γ*2 expression is restricted to adipose tissue, where it acts as a master transcription factor in adipogenesis: *in vitro *it promotes adipocyte differentiation, while *in vivo *it lowers circulating NEFA and improves whole-body insulin sensitivity [35]. PPAR*γ*1 is more broadly expressed; it is abundant in adipose tissue, macrophages, and gastrointestinal epithelium where it cooperates with the transcription factor Hic5 to promote epithelial cells differentiation during embryonic development [34, 36–38]. This observation suggests that PPAR*γ* is involved in the differentiation of several epithelia, including colon epithelium.

Regulation of gene expression by PPAR*γ* occurs through distinct mechanisms. In the absence of agonists, the PPAR*γ*/RXR heterodimer represses gene transcription by stabilizing its interactions with the corepressor complexes at the promoter region of target genes. Ligand binding enables the recruitment of coactivators to promote gene transcription. A recently disclosed mode of action, called transrepression, involves gene repression in a ligand-dependent manner through protein–protein interactions with NF*κ*B, AP1, Smads, STATs, and NFATs [40, 41]. Specifically, when activated by TZD, PPAR*γ* inhibits the expression of several inflammatory genes in macrophages with beneficial effects, as, for instance, in inflammatory bowel diseases [42, 43]. This is attained through the recruitment and stabilization of the N-CoR complexes at the NF*κ*B responsive promoters of proinflammatory genes by a functionally distinct pool of PPAR*γ* susceptible to ligand-dependent SUMOylation at lysine 365 (Figure 2) [44]. The metabolic and anti-inflammatory properties of PPAR*γ*, along with its role in cell differentiation, have encouraged to pursue for new functions in cancer (Tables 1 and 2).

## 3. *PPAR* **γ** and Cancer 

PPAR*γ* is expressed in a variety of tumors and its role in cancer formation/progression has been controversial for long time [45–52]. *In vitro *studies have shown that PPAR*γ* activation results in growth arrest of epithelial-derived cancer cell lines, including those from thyroid, lung, prostate, breast, pituitary, and colon [53–58]. Consistently, some PPAR*γ* downstream targets, such as the CDK inhibitors p18, p21, and p27, are induced determining a cell cycle block [59, 60]. The tumor suppressor gene, PTEN, is also upregulated upon PPAR*γ* activation in different cell lines, inhibiting PI3-kinase and AKT phosphorylation, hence reducing cell migration and proliferation [61–64]. Tumor growth is also inhibited through the interference with the APC/*β*-catenin and COX-2/PGE2 signaling pathways, which are pivotal in colon carcinogenesis [8, 65]. PPAR*γ* downregulates matrix metalloproteinase-7 (MMP-7) and induces MMP inhibitors expression, suppressing tumor cell invasion [66, 67]. In colon cancer and non-small-cell lung carcinoma cells, PPAR*γ* induces the expression of the transcriptional repressors TSC22 and GADD153, respectively [68, 69]. To reinforce the anti-proliferative effects, PPAR*γ* downregulates the anti-apoptotic protein Bcl-2 [70]. More recently, PPAR*γ* has been endowed with an antiangiogenic activity through inhibition of VEGF and its receptors in different cells [71, 72]. As previously mentioned, PPAR*γ* inhibits NF*κ*B-mediated gene transcription. Constitutive activation of NF*κ*B is frequently observed in solid tumors leading to overexpression of a variety of target genes that confer growth advantages and resistance to chemotherapy [73, 74]. Finally, PPAR*γ* hampers the Epithelial–mesenchymal transition (EMT), a well-known process that allows cancer cells to acquire invasive ability, a prerequisite for metastasis formation. EMT is characterized by a reversible conversion of polarized epithelial cells into highly motile fibroblastoid cells accompanied by loss of cell–cell adhesion molecules, such as E-cadherin, downregulation of epithelial differentiation markers, and expression of mesenchymal markers such as vimentin and N-cadherin [75, 76]. PPAR*γ* inhibits TGF*β*-induced EMT in lung and pancreatic adenocarcinoma cell lines by antagonizing Smad3-dependent transcriptional activity. As a result, EMT, morphological changes, MMPs secretion, migration, and invasion are greatly diminished [76]. All together, these data strongly support a role for PPAR*γ* as tumor suppressor; in contrast, a few studies have provided evidence that it acts as a tumor promoter [14, 64, 77, 78]. In line with this latter hypothesis, PPAR*γ* expression has recently been found elevated at the mRNA and protein levels in chicken embryo fibroblasts (CEFs) transformed by the Ski oncogene. These cells, unlike most other oncogene-transformed cells, do not display the classical Warburg effect and have a reduced glucose utilization associated with increased fatty acids *β*-oxidation. PPAR*γ* upregulation appears then to drive the oncogenic lipid metabolism required for high-rate cell proliferation and enhanced survival. PPAR*γ* knocked-down by RNA interference reverses the expression of both PPAR*γ* and its target genes [79]. Another study has showed that HER2-overexpressing breast cancer cells present an increased PPAR*γ* expression that exacerbates tumor development as it fuels lipogenic enzymes reducing accumulated fatty acids toxicity. Her-2 transformed cells have adopted an oncogenic lipid metabolism that is instrumental in cell proliferation and survival. Her-2 overexpression significantly activates the MAPK pathway responsible for most of the effects observed. However, this pathway negatively regulates PPAR*γ*, so trastuzumab administration not only reduces the levels of MAPK activity but also downregulates PPAR*γ*; these beneficial effects appear more remarkable when combined with PPAR*γ* agonists [80]. A more recent work, finally, suggests for PPAR*γ* a dual role as a tumor-promoting factor in neuroblastoma cells and as a tumorsuppressor in breast cancer cells. In the former case, PPAR*γ* induces cell growth *in vitro *and tumor growth in mouse xenografts through the induction of inflammation and of NHE1, an oncogenic factor. Conversely, in the latter case, it inhibits NHE1 expression. These discordant results have been attributed to cell type-specific differences in the regulation of NHE1 and other target genes [81]. Collectively, the large wealth of data accumulated so far on the specific role that *PPARG* plays in tumorigenesis supports a cell growth restraining function, hence a tumor repressor activity.

Since PPAR*γ* is expressed in differentiated epithelial colonic cells and in colorectal cancer (CRC), a specific role has been hypothesized in colon pathophysiology [38, 82]. CRC is one of the most frequent malignancies in western countries and a common cause of cancer-related death worldwide [83]. A great effort has, thus, been made to understand the molecular mechanisms through which *PPARG* affects CRC progression. The lack of suitable cellular models to assess its role in normal colonic epithelial cells has made necessary to evaluate the effects of PPAR*γ* agonists *in vivo*. Indeed, the first two articles reported that administration of PPAR*γ* ligands increased the incidence of colon tumors in Apc+/Min mice [84, 85]. In contrast, PPAR*γ* produced no effects on tumor incidence in Apc/1638N and 1309 mice, using both genetic and pharmacological models [37, 86]. A more recent study has shown that pioglitazone, a TZD family member, suppresses colon tumor growth in Apc+/Min mice [87]. These contradictory observations on the *Pparg* role in tumorigenesis have apparently been resolved by more recent data obtained by a tissue-specific *Pparg* biallelic knockout in Apc+/Min mice. In this strain, an increased tumor incidence and tumor size is observed, consistent with the *in vitro *data obtained in human cancer cell lines: PPAR*γ* ligands inhibit cell growth even in the presence of *APC *mutations [88–91]. In azoxymethane (AOM)-treated rodents, the most widely used preclinical model of sporadic CRC in rodents, *Pparg* inhibits colon carcinogenesis [92–94]. In this system, TZDs act as potent suppressors of tumor formation. Of note, some of the effects attributed to TZDs can be due to PPAR*γ*-independent effects [95, 96]. A direct role of PPAR*γ* as tumor suppressor is confirmed by the observation that hemizygous *Pparg* colon-specific knockout mice display a significantly higher incidence of colon tumors following AOM treatment [97]. Epidemiological studies in humans have clearly established a link between chronic inflammatory conditions and tumor initiation. Inflammatory bowel diseases (IBD) are associated with a higher risk of development of a CRC subtype known as colitis-associated cancer (CAC). In these cases, tumor promotion is mainly due to the presence of leucocyte infiltration and inflammatory mediators. Consistently, administration of nonsteroidal anti-inflammatory drugs to IBD patients results in reduced CRC development [98]. In mouse models, PPAR*γ* activation by selective agonists has been shown to attenuate the severity of chemically induced IBD also in colon-specific *Pparg*-null mice [99]. This is due to activation of PPAR*γ* in macrophages, central orchestrators of the inflammatory response in IBD. In agreement, *Pparg* ablation in these cells increases the susceptibility to chemically induced colitis, suggesting that PPAR*γ* can inhibit the inflammation-associated tumor initiation acting both in epithelial cells and in macrophages [99, 100]. In spite of the results obtained in murine models, evidence of PPAR*γ* involvement in human colon carcinogenesis is still circumstantial. *PPARG *is expressed at high levels in about 60% of sporadic human CRCs. Specific loss-of-function mutations have been reported in 8% of primary CRCs, an observation not confirmed in a subsequent study, in which these mutations were defined as “very rare events” [101, 102]. Increasing evidence suggests that PPAR*γ* activity is attenuated during the transition from adenoma to carcinoma, likely explaining why PPAR*γ* agonists can block the early stages of tumorigenesis. In fact, they inhibit aberrant crypt focus (ACF) formation but have little or no effect on advanced tumor stages [37]. PPAR*γ* attenuation may involve, at least in part, its phosphorylation operated by the mitogen activated kinase ERK 1/2, and its ligand-independent SUMOylation, two posttranslational modifications that negatively regulate PPAR*γ* activity [103, 104]. Loss-of-function mutations and the reduced activity due to posttranslational modifications, however, do not fully explain the low *PPARG* expression found in 35% of sporadic CRCs [105]. Interestingly, these levels have been associated with a more aggressive course, EMT activation, and patients' worse prognosis, indicating that *PPARG *can be considered an independent prognostic factor [105, 106]. Other mechanisms should be suggested to explain the low *PPARG *levels observed.

## 4. Epigenetics and Cancer

It is well accepted that genetic mutations as well as epigenetic modifications contribute to tumor establishment and/or progression. “Epigenetics” indicates changes in chromatin structure that result in different gene expression patterns without alterations of the primary DNA sequence and regardless of heritability [107]. In contrast to genetic lesions, epigenetic variations are reversible and involve changes in DNA methylation, histone posttranslational modifications, and expression of noncoding RNAs (ncRNAs) [108]. Over 25 years ago, Feinberg and Vogelstein found an extensive loss of DNA methylation in colon cancer cells. This global hypomethylation has been associated with increased genome instability and overexpression of a variety of genes implicated in CRC pathogenesis [109]. More recent findings indicate that the association of a global hypomethylation with a discrete hypermethylation at promoter regions of specific genes involved in cell-cycle regulation, DNA repair, apoptosis, angiogenesis, adhesion, and invasion is a common event in tumorigenesis [110]. Promoter hypermethylation at *MLH1, APC, RB1, VHL, MGMT, GSTP1,* and *BRCA1 *represents paradigmatic cancer-related epigenetic silencing events [111]. Available data support the notion that epigenetic abnormalities arise in the earliest steps of tumor development. Aberrant methylation patterns are, in fact, already recognized in preneoplastic lesions such as dysplastic ACFs and hyperplastic polyps and are considered as a risk factor for the development of CIMP-positive CRCs [110–114].

Four DNA methyltransferases (DNMTs): DNMT1, DNMT3A, DNMT3B, and DNMT3L establish and regulate the global patterns of DNA methylation in healthy and tumor cells. DNMT1 associates with S-phase replication foci and acts primarily as a maintenance methyltransferase. DNMT3A and DNMT3B are essential for *de novo *methylation during embryonic development. Finally, DNMT3L forms a complex with DNMT3A and DNMT3B in embryonic stem cells and stimulates their activity [115–117]. Deregulation of DNMTs expression contributes to tumorigenesis, conferring an aberrant methylation pattern and causing tumor suppressor genes promoter methylation [118, 119]. DNA methylation alone, however, is not sufficient to repress gene transcription. A complex and intertwined set of posttranslational modifications of the core histone tails dynamically imparts either repressive or activating transcriptional signals, following the so-called histone code. These marks, and the cellular machinery regulating them, are also frequently disrupted in cancer [119].

Methylation at lysine 9 of histone H3 (H3K9) is one of the most studied histone modifications, and SUV39H1 was initially recognized as endowed with H3K9 histone methyltransferase (HMTs) activity. Recently, more H3K9 specific methyltransferases have been identified: SUV39H2, G9a, SETDB1, and EuHTMase1, each of them able to cause different methylation states. G9a is mainly responsible for SUV39H1 mono- and dimethylation primarily found in euchromatin, while SUV39H1 directs the trimethylation of the same residue found in facultative or constitutive heterochromatin [119–122]. SUV39H1, in addition, is overexpressed in tumors [122].

Trimethylation at lysine 27 of H3 (H3K27me3) is a distinct histone modification primarily involved in the maintenance of gene silencing. Enhancer of zeste 2 (EZH2) is the unique histone methyltransferase with H3K27 substrate specificity. EZH2, together with EED and SUZ12, forms the polycomb group (PcG) repression complex 2 (PRC2) and initiates gene silencing by trimethylating H3K27 and recruiting the PRC1 complex. This latter includes BMI-1, RING1, HPC, and HPH and its binding to the DNA blocks the recruitment of activating transcriptional factors, such as SWI/SNF, and prevents initiation of transcription by RNA polymerase II [123].

Acetylation of lysine 27 of H3 and lysine 16 of H4 (H3K27ac and H4K16ac), in contrast, characterize the promoter regions of actively transcribed genes. Also di- and trimethylation of lysine 4 of H3 histone (H3K4me2/me3) are active marks, at odds with other methylated residues. H3K4 methylation seems to protect gene promoters from *de novo *DNA methylation in somatic cells, preventing the recruitment of heterochromatin-inducing proteins [124]. These “activating” histone marks are carried out by a series of histone acetyltransferases (HATs), among which the best known is CBP/p300 and the associated pCAF, and by specific K4H3 methyltransferases as MLL and ASH [113, 125, 126]. The “active” histone modifications are frequently altered in cancer cells, in line with the fact that histone deacetylases (HDACs), that remove histone acetylation, are overexpressed or mutated in different tumor types [127]. DNA methylation and histone modifications are strictly interconnected. Genome-wide DNA methylation profiles, in fact, suggest that DNA methylation is better correlated with histone methylation patterns than with the underlying genome sequence context. Specifically, DNA methylation is correlated with the presence of H3K9 methylation and the absence of H3K4 methylation [128, 129]. The relationship between DNA methylation- and EZH2-dependent (PRC2-dependent) silencing has not been completely elucidated. In prostate cancer cells, these two epigenetic modifications act in an independent and inverse mode; conversely, in CRC cell lines DNA methylation is accompanied by H3K27me3 formation [123, 130]. This discrepancy is explained by tissue- and cancer-specific differences related to activation of specific silencing pathways [123]. Cooperation of DNA methylation and histone modifications requires proteins that directly readout the DNA methylation pattern and recruit the histone modifying enzymes or vice versa. In some cases, the DNMTs, such as DNMT3b, directly interface with the histone methylases SUV39H1, EZH2, and G9a [128–130]. Accessory proteins are, in other cases, required for recruiting histone-modifying enzymes and/or DNMTs. Methyl-DNA binding proteins (MBP) form a protein family whose members recognize 5′meC, and some of them are endowed with lysine methyltransferase activity to repress transcription through heterochromatin formation [131]. MeCp2 binds a single methylated CpG dinucleotide and recruits HDACs to silence transcription via histone deacetylation; moreover, it plays a role in tumorigenesis and has been shown to target several genes in different tumors *in vivo *[132, 133]. Kaiso, another zinc finger domain containing protein, is capable of binding not only a pair of methylated CpG dinucleotides but also unmethylated DNA. Given Kaiso's ability to repress transcription at both methylated and unmethylated promoters, it is currently difficult to assess how important its mCpG binding role is in cancer [132]. Finally, the SRA-domain containing proteins, UHRF1 and UHRF2, recruit HDAC1 and methylate tumor suppressor gene promoters. UHRF1 has affinity for hemimethylated DNA and recruits DNMT1 to ensure the epigenetic inheritance of DNA methylation and maintenance of histone marks [131, 133]. Additionally, UHRF1 seems to play a role in the DNA damage response and is able to recruit the *de novo *DNMTs on gene-specific promoters [131, 133–135]. Finally, in addition to proteins, also RNAs can modulate chromatin structure. Long non-coding and micro RNAs are widely transcribed in the genome, and their roles are only at the beginning to be understood. Recent studies suggest that some of them can function as an interface between DNA and specific chromatin remodelling activities. However, their involvement in human cancers has not been fully elucidated yet [136, 137].

In this scenario, the CIMP (CpG island methylator phenotype) positive tumors that account for approximately 20% of CRCs deserve special attention [114]. They are characterized by promoter hypermethylation of specific genes, defined “CIMP markers”, and by microsatellite instability (MSI). This condition of genomic instability is in contrast with CIN-positive (chromosomal instability) tumors characterized by a different genomic status that drives the adenoma/carcinoma events. Activating *BRAF *mutations also characterize CIMP+ tumors that likely arise from serrated polyps typically located to the right colon [108, 114]. Although the exact mechanisms underlying the aberrant DNA methylation of these tumors remain to be clarified, current evidence suggests that the CIMP phenotype may be an early, possibly tumor initiating event [115].

## 5. *PPARG *Epigenetic Regulation 

**Figure 3:**
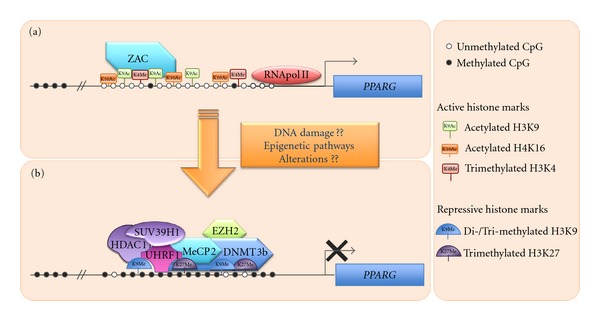
Schematic drawing of the proposed mechanism(s) involved in *PPARG* silencing. (a) In PPAR*γ*-expressing cells, the unmethylated or partially methylated core promoter is bound by still unknown transcriptional factors, among which only the zinc-finger protein ZAC has been identified. Active histone marks, such as H3K9ac, H3K4me3 and H4K16ac are present together with the RNA polimerase II. (b) Upon unknown stimuli, the *PPARG* promoter becomes hypermethylated, enriched in H3K9me2, H3K9me3 and H3K27me3. Furthermore, a repressive complex containing UHRF1, MeCP2, DNMT3b, HDAC1, SUV39H1 and EZH2 is recruited inducing transcription repression. Partially adapted from reference [39].

Consistent with the results reported above, research efforts have been made to investigate the mechanisms that regulate *PPARG *expression. At the moment, we have only a limited knowledge of the transcription factors and mechanisms that modulate *PPARG*.* Pparg*2 promoter demethylation has recently been shown to correlate with 3T3-L1 adipocyte differentiation. Interestingly, MeCP2 is associated with a silenced and methylated *Pparg*2 promoter in undifferentiated preadipocytes [138, 139]. In line with this, MeCP2 is recruited onto the *Pparg*1 promoter along with HP1, H3K9me3, and EZH2-dependent H3K27me3, driving its epigenetic silencing in hepatic stellate cells (HSCs). This event is pivotal in inducing HSCs transdifferentiation into myofibroblasts leading to a fibrotic liver [39, 140]. More recent studies from our laboratory have addressed the epigenetic regulation of *PPARG* transcription in human colon cancer [141]. The analysis of CRC cell lines has demonstrated that *PPARG* promoter hypermethylation correlates with reduced gene transcription, presence of H3K9me3, and H3K27me3 and concomitant recruitment of HDAC1, MeCP2, and EZH2. Conversely, epigenetic treatment with 5-aza-2′-deoxycytidine and trichostatin A induces *PPARG* reexpression associated with recruitment of active histone marks, RNAPol-II, and the transcriptional activator ZAC. Remarkably, the same promoter region that is methylated in *PPARG*-negative cells is methylated also in 80% of our *PPARG*-negative CRCs. These data provide the first direct evidence that *PPARG* is epigenetically downregulated in human CRC, and this condition is associated with poor patients' prognosis [141]. In addition, we have identified UHRF1 as a mediator of *PPARG* silencing. The UHRF1 ability to keep promoters in a hypermethylated state, together with the possibility of mediating *de novo* methylation, gives this factor an important role in cancer development through silencing tumor suppressor genes. UHRF1 upregulation is inversely correlated with *PPARG* expression in an advanced tumor stages CRC subgroup [142]. Consistently, UHRF1 knockdown *in vitro *reactivates *PPARG*, while UHRF1 overexpression induces its repression due to recruitment of MeCP2, EZH2, and DNMT3b. The histone methyltransferase SUV39H1 is also a constituent of this multiprotein repression complex (Figure 3). UHRF1, DNMT3b, and SUV39H1 are frequently upregulated in CRC, and our studies confirm their role in the epigenetic repression of protein-coding genes [118, 122, 141, 142]. Altogether, these results suggest that epigenetic mechanisms play a crucial role in *PPARG* deregulation and tumor development. *PPARG* epigenetic silencing might, thus, be a critical and common step of the tumorigenic process. The association with UHRF1 activation, especially in advanced tumor stages, suggests that they are part of a more complex regulatory circuit. In agreement with these results, a very recent methylation profile carried out in supratentorial and spinal ependymomas suggests that epigenetic silencing of tumor suppressor genes, including *PPARG*, is crucial in the development of these neoplasms [143].

## 6. Concluding Remarks

The recent achievements in the understanding of the tumorigenic mechanisms have clearly demonstrated that epigenetic deregulation can result in altered gene function and malignant cellular transformation. Genomic instability is emerging as a hallmark of cancer and the rapidly evolving field of cancer epigenetics has led to the identification and characterization of the CIMP phenotype with the provocative hypothesis of epigenomic instability. In this context, it has been suggested that an incorrect association between nuclear receptors and the epigenetic machinery may contribute to tumor development. Among nuclear receptors, PPARs are pivotal in several biological processes. Specifically, *PPARG* has been shown to protect against tumor progression. Epigenetic silencing is emerging as an unprecedented level of complexity of *PPARG* expression. Deciphering the precise code that dictates this event and the players involved is one of the major present efforts. This approach will provide useful insight as to how epigenetic events at *PPARG* are related with the genomic instability status in CRC. It will also address the unanswered question as to whether *PPARG* epigenetic deregulation contributes to the establishment of “precancerous lesions” or very early cancer developmental stages. Lastly, it will provide the basis for designing more efficient epigenetic drugs affecting cancer initiation/progression. *PPARG* can then be viewed as a target of novel therapeutic strategies.

## Figures and Tables

**Table 1:** Effects of PPAR*γ* expression/activation in different human tumors.

Tumor type	Supposed function	References
Colon cancer	Associated with good patients' prognosis	[105, 106]
Hepatic cancer	Protective effect against cancer	[59]
Renal cell carcinoma	Potential target of pharmacological therapy	[45]
Prostate cancer	Potential target of pharmacological therapy	[45]
Ovarian cancer	Protective effect against cancer	[46]
B cell lymphoma	Potential target of pharmacological therapy	[104]
Ependymoma	Not defined	[143]
Breast cancer	Protective effect against cancer	[47, 50, 51]
Neuroblastoma	Protective effect against cancer	[52]
Pancreatic Cancer	Associated with shorter overall survival	[77]
Thyroid carcinoma	Protective effect against cancer	[48]
Liposarcoma	Induction of tumor differentiation	[49]

**Table 2:** Effects of TZD administration on human cell lines derived from different tumors.

Cell types	Observed effects	References
Colorectal cancer	Growth arrest, differentiation, apoptosis	[39, 57, 67, 68, 70]
Thyroid carcinoma	Growth arrest	[54]
Prostate cancer	Growth arrest	[55]
Ependymoma	Growth arrest	[39]
Lung carcinoma	Cell cycle arrest	[58, 62, 69, 76]
Breast cancer	Cell cycle arrest	[56, 66]
Hepatoma	Cell cycle arrest	[59, 63]
Neuroblastoma	Increased proliferation	[81]
Pancreatic carcinoma	Cell cycle arrest	[60, 61]
